# Spread of gambling abstinence through peers and comments in online self-help chat forums to quit gambling

**DOI:** 10.1038/s41598-022-07714-2

**Published:** 2022-03-07

**Authors:** Kenji Yokotani

**Affiliations:** Graduate School of Sciences and Technology for Innovation, Tokushima University, 1-1, Minamijosanjimacho, Tokushima-shi, Tokushima 770-0814 Japan

**Keywords:** Human behaviour, Public health

## Abstract

Habit formation occurs in relation to peer habits and comments. This general principle was applied to gambling abstinence in the context of online self-help forums to quit gambling. Participants in this study, conducted between September 2008 and March 2020, were 161 abstinent and 928 non-abstinent gamblers who participated in online self-help chat forums to quit gambling. They received 269,317 comments during their first 3 years of forum participation. Gamblers had an increased likelihood of 3-year continuous gambling abstinence if they had many peers in the forums. However, they had a decreased likelihood of gambling abstinence if they received rejective comments from the forums. Based on these results, online social network-based interventions may be a new treatment option for gamblers.

## Introduction

People develop habits within the context of their peers’ habits and comments. For example, those whose peers smoke are at an increased risk of smoking^1^, and their peers’ positive comments on smoking can also encourage their smoking habits^2^. In the current study, this general principle of habit formation was applied to gambling abstinence in the context of online self-help forums to quit gambling. Gambling is practiced worldwide, with 22.6% of European adolescents gambling. Additionally, those who gamble have increased risks for substance use and truancy at school^3^. People with problematic gambling habits also have an increased risk of committing income-generating offenses^4,5^. Furthermore, their gambling habits are affected by their peers’ habits^6^ since they have many peers who gamble excessively^7^ and participate in many online gambling groups^8^. These findings indicate that their problematic gambling habits are associated with habits influenced by their peers online and offline. Although online gambling group could be linked to problem gambling^8^, online groups to quit gambling could be linked to gambling abstinence^9^. Hence, the current study focuses on the latter and examines the links between individuals’ gambling abstinence and the abstinence and comments of their peers on online social networks. To clarify these links, I used the social network analysis, a common method for analyzing social relationships^10^. Clarification of these links could help create an online social network-based approach^11,12^ to treat people with gambling disorders and reduce the impact of the social problems they cause^5,13^.

This study uses complex contagion theory to explain habit formation^14^. This theory assumes that an individual will adopt a new habit from others when they experience multi-layered rather than a single exposure to that habit^15^. For example, people are likely to integrate a new form of technology into their lives when they have seen multiple friends using it and hear their opinions on it^14^. Complex contagion theory has been used to explain physicians’ adoption of new types of medical treatment^16^, acceptance of new music among mobile app users^17^, and fake news among the general population^18^.

According to the contagion theory, people’s habits are affected by those of their peers. For example, in a previous study, participants’ daily volumes of sugar intake and physical exercise were predicted by those of their peers. Those who frequently met peers with a high sugar intake habit had a high risk of eating a high-sugar diet, whereas those who frequently met peers with an exercise habit had a higher chance of engaging in exercise^19^. Similarly, other studies have shown that those who are surrounded by obese peers are more prone to obesity^20^. Conversely, those who are surrounded by peers who play soccer and engage in jogging are more likely to perform these activities themselves^11,21^. Even when Air Force Academy recruits were randomly grouped to live together in squadrons and eat the same meals, recruits assigned to squadrons with poor exercise habits and physical performance had worse physical performance than those without^22^. Since several randomized controlled trials did not confirm peers’ positive effects on exercise^23–25^, these trials suggested that peers’ procrastinate habits affect the participants’ habits.

People’s habits were also affected by their peers’ comments. In a previous study, smokers were more likely to talk positively about smoking, whereas nonsmokers were more likely to speak of it negatively^26^. Gunther et al. (2006) found that peers’ positive comments about smoking fostered participants’ smoking habits, whereas peers’ negative comments hindered them. The causal relationship between peers’ negative comments about smoking and smoking abstinence has also been confirmed in several randomized controlled trials^27–30^. For example, fifty-nine junior high schools were randomly divided into experimental and control clusters. In the experimental cluster, 17.5% of junior high school students expressed negative comments about smoking to their peers, while those in the control cluster did not receive any such intervention^31^. The experimental cluster had a lower smoking rate in all junior high schools, and this effect was confirmed 1 and 2 years later^31^. These randomized controlled trials indicate that peers’ negative comments on smoking habits decrease individuals’ chances of adopting these habits.

The social contagion of habits via peers and their comments has also been confirmed in online social networks^32^. In a previous study, members who actively engaged in an online self-help group to quit smoking felt connected to other members and received supportive messages from them^33^. Peers and supportive messages have been shown to enhance individuals’ self-efficacy regarding quitting smoking^33^ and predict smoking abstinence^34^. Similarly, in another study, individuals’ active engagement in self-help groups predicted smoking abstinence^35^. Group registration and chats have also been shown to enhance individuals’ chances of abstaining from smoking^36^. One randomized controlled experiment reported that adolescents who joined an online group to quit smoking smoked less often than those who did not^37^. In another randomized controlled experiment, a group that saw online comments encouraging smoking reported less willingness to quit smoking than a group that saw comments denigrating it^38^. These two randomized controlled trials imply that online peers’ abstinence habits positively influence individuals’ abstinence habits, but negative comments about abstinence negatively influence their habits^39^.

Given that previous findings have been in the context of other addictive disorders (e.g., smoking), the contagion theory should also be relevant to online self-help forums to quit gambling^40^. Regarding the existing literature in this regard^41^, although one study found positive links between gamblers’ comments in online self-help forums and their abstinence^9^, the links between their peers’ abstinence and comments with their own abstinence were not examined. Hence, this study tested these links in the context of online self-help forums to quit gambling.

This study also explores differences in social contagion of gambling abstinence across comment categories because many studies have implied that these are not necessarily linked to gambling abstinence in the same way^41–43^. Particularly, rejective comments from others make it difficult for gamblers to continue gambling abstinence^44,45^. This study also regarded individuals who had been continuously abstinent for 3 years as more robust abstinent individuals than those who had been abstinent for 90-day periods^46,47^. Although several studies have considered individuals with 90-day abstinence periods^48^, overall, the trend is toward the use of data related to gamblers with 1-year continuous abstinence periods^49,50^. Based on these findings, gamblers with at least 3 years of continuous abstinence were regarded as abstinent gamblers in this study. In line with this, the influence of peers within the online self-help group on quitting gambling was limited to the first 3 years after the gambler joined the self-help group.

This study has two hypotheses:

### H1

Gamblers are more likely to be abstinent if they have many peers in self-help forums to enable them to quit their gambling.

### H2

Gamblers are less likely to be abstinent if they receive rejective comments from their peers in the forums.

## Methods

### Data source

**Figure 1:**
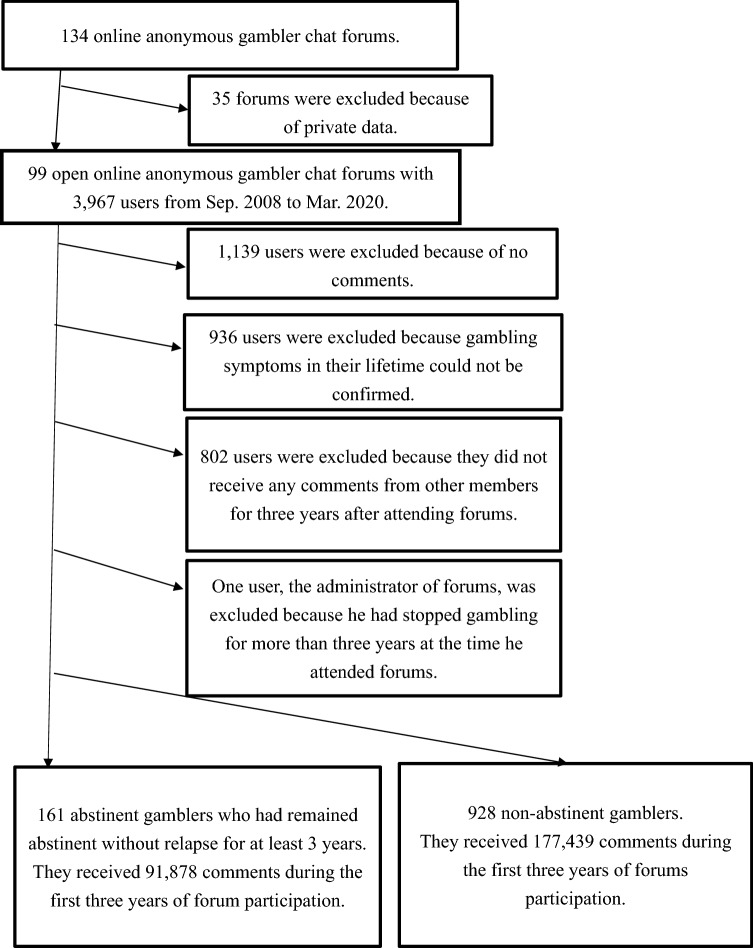
Flow of participants.

This study used online registry data, parts of which were used in another study^9^. I web-scraped the website of online self-help chat forums to quit gambling in Japan^51^. This website does not prohibit web scraping in its terms of use^51^. To avoid overloading the server of this website^52^, a one-second waiting period was added when moving through the pages of the site. To legally download data from this website^53^, I downloaded data only from forums that I could browse freely and not from forums that required a password. Post notifying the administrator of the forums’ academic use, I found 134 forums on the site, but 35 of them required a password. Therefore, I excluded them and downloaded data from the remaining 99 forums (Fig. 1). The website is mainly for online forums. However, it has a few offline forums in Osaka, Tokyo, or Sapporo. The forums also recommended anonymous names rather than real names for chatting, but the forums had no connection with anonymous gamblers^50^. Notably, browsing the forums is free, but posting on these forums requires prior approval from the administrator. Individuals who are gamblers themselves or have a family member who is a gambler are allowed to post on these forums. The forums were conducted by gamblers without outside experts or strict rules. The forums were divided into those for users aiming for 1 week, 1 month, 3 months, 6 months, 1 year, 3 years, and more than 3 years of abstinence and were attended according to the number of days of abstinence. However, a user aiming for 6 months of gambling abstinence could attend a forum for those aiming for 3-month gambling abstinence and vice versa. In addition to daily and anniversary reports (such as 300 abstinent days), there were also forums for chatting. Moreover, these forums are always open and free to join, and the time and duration of participation vary greatly from user to user. The forums ranged from several days to months. When a forum exceeds 5000 posts, it ends, and a new forum begins.

### Sample size

Few studies have focused on gamblers who have been abstinent for 3 years continuously^49^. Therefore, the ideal sample size was determined by referring to a study of smokers who had been abstinent for 3 years^46^. This was based on the fact that smokers and gamblers have similar biological factors^54^. For example, an increase in dopamine neurotransmission in the mesocorticolimbic regions has been observed in both smoking^55^ and monetary reward^56^ situations. Both regular smokers^57^ and problem gamblers^58^ inhibit monoamine oxidase in their brains. In Richmond et al.’s (1997) study, 13.8% of smokers achieved 3-year abstinence from treatment, while 86.2% did not. Based on these results, the allocation ratio between abstinent and non-abstinent gamblers, that is, the ratio of abstinent and non-abstinent gamblers as the numerator and denominator, respectively (13.8/86.2), was set at 0.16. In this study, 440 participants were required to achieve satisfactory power (0.95) with an alpha of 0.05 for medium effect size.

### Participants

Users in the forums posted texts in Japanese between September 8, 2008, and March 10, 2020 (Fig. 1). These 99 forums contained 390,406 posts. Since these posts contained many long sentences, they were divided into units of two sentences. Further, these units of two sentences were counted as comments. If the username of another user, B, is entered in one of the comments of user A, I regarded that user B receives a comment from user A. If user A makes a long post with 50 comments (100 sentences), and for each of the 50 comments, the username “B” appears, then the B received 50 comments from A. However, if the username "B" only appears in the first comment, then B receives only one comment from A.

Among the 99 forums that were freely available for browsing, 3967 users were identified based on their usernames (Fig. 1). Among them, 1139 users were excluded because their postings contained less than three words or only advertisements using "http." Furthermore, 936 users were excluded because their posts did not reveal gambling symptoms over their lifetime (the assessment of gambling symptoms is presented in the next section). Furthermore, 802 users were excluded because they had not received any comments from other members during the 3 years of forum participation. Additionally, a user who served as the forum administrator was excluded because he had been abstinent gamblers before attending the forum. The final participants were 1089 gamblers who experienced at least one gambling symptom in their lifetime, received at least one comment from their online peers, and were not abstinent at the time of their first forum attendance, although some of them became abstinent after 3 years. By limiting the comments they received to the first 3 years of their participation and excluding all subsequent comments, this study sought to clarify what types of comments and peers made people more likely to become abstinent gamblers after 3 years.

### Basic data collection

#### Gambling symptoms

Among the users’ comments, text was extracted based on keywords related to 10 gambling symptoms: one gambling symptom was an illegal behavior associated with gambling^59^, and nine symptoms of gambling disorder were based on the fifth edition of the Diagnostic and Statistical Manual of Mental Disorders^40^. For example, “lie” is a keyword used for examining the symptom of lies associated with gambling; therefore, all text involving “lie” was extracted. Two raters, who majored in clinical psychology as undergraduate students and received two-hour training regarding gambling disorders from a Japanese clinical psychologist^5^, independently read the text and evaluated whether or not the user had experienced the aforementioned 10 symptoms in their lifetime. To check the validity of the raters’ evaluations, the psychologist blindly evaluated randomly selected texts from 100 participants. Kappa coefficients confirmed that the psychologist and two raters were primarily in “almost perfect or perfect agreement”^60^ in their evaluation of the presence of gambling symptoms (0.835, 0.860; Supplementary Table 1). Based on their assessment, individuals with at least one gambling symptom in their lifetime were included as final participants (Fig. 1). The number of gambling symptoms and gambling symptoms for which agreement between the psychologist and the two raters was above substantial (0.60)^60^, are used for the following analysis.

#### Demographic variables

Gender and age were estimated using the first 1000 words of the aforementioned gamblers because these words frequently involved self-introduction statements. The gender and age of one-third of the gamblers were determined using a Japanese application^61^ (Table 1).

**Table 1:** Differences in demographic variables, gambling problems, peers, and received comments between abstinent and non-abstinent gamblers in the first 3 years of attending the online self-help forums to quit gambling.

*Demographic variable*	Abstinent gamblers n = 161		Non-abstinent gamblers n = 928				
	*M*	*S.E*	*M*	*S.E*	*t*	*d*	*p*
Age (years)	33.800^a^	0.061	36.180^e^	0.011	− 2.075	− 0.26	*
Male rate	0.852^b^		0.844 f.		0.230	0.02	
*Gambling problem*							
Total amount of debt (million yen)	4.580^c^	0.194	2.163g	0.017	1.364	0.54	
Duration of gambling (years)	12.700^d^	0.074	11.211h	0.014	1.296	0.21	
Number of symptoms (min: 1, max: 10)	3.124	0.006	2.720	0.001	2.556	0.23	*
Gambling tolerance	0.453	0.002	0.374	0.000	1.873	0.16	
Gambling withdrawal	0.193	0.001	0.177	0.000	0.47	0.04	
Unsuccessful control over gambling	0.733	0.001	0.749	0.000	− 0.424	− 0.04	
Preoccupation with gambling	0.689	0.001	0.553	0.000	3.411	0.28	***
Gambling as problem avoidance	0.155	0.001	0.158	0.000	− 0.10	− 0.01	
Chasing one’s gambling loss	0.112	0.001	0.142	0.000	− 1.11	− 0.09	
Lies associated with gambling	0.348	0.001	0.208	0.000	3.501	0.34	***
Loss of relationships and opportunities	0.292	0.001	0.231	0.000	1.59	0.14	
Reliance on others to provide money	0.143	0.001	0.087	0.000	1.905	0.19	
Illegal acts for gambling	0.006	0.000	0.041	0.000	− 3.861	− 0.19	***
*Characteristics of peers in forums*							
Number of peers who commented	47.981	0.173	22.543	0.015	5.635	0.75	***
Number of senior abstinent peers who commented	8.752	0.076	5.431	0.010	1.64	0.16	
*Characteristics of comments received during forums*							
Number of received comments	570.671	3.538	191.206	0.281	4.139	0.58	***
Number of change comments	25.342	0.144	9.560	0.011	4.241	0.60	***
Number of sustain comments	2.839	0.019	1.540	0.003	2.583	0.51	*
Number of general comments	207.882	1.404	70.588	0.119	3.764	0.25	***
Number of acceptive comments	93.429	0.551	30.211	0.042	4.436	0.64	***
Number of rejective comments	15.720	0.108	8.776	0.014	2.417	0.64	*
Number of neutral comments	225.460	1.387	70.530	0.100	4.325	0.25	***

The data sizes of the demographic variables and gambling histories were smaller than those of the comments. S.E: Standard Error*,* ^a^n = 75, ^b^n = 115, ^c^n = 19, ^d^n = 50, ^e^n = 428, ^f^n = 634, ^g^n = 107, ^h^n = 251. One million yen was approximately US$ 9193.* *p* < 0.05, **p < 0.01, ***p < 0.001.

#### Gambling history and debt

Among the gamblers’ comments, text was extracted based on the keywords “years” and “history.” The text was then read by the two raters. If the text revealed a gambling history, users’ years of gambling were recorded (Table 1). Similarly, text was extracted based on the keywords “cash,” “yen,” “ten thousand,” and “borrow.” If the text indicates debt, the debt is listed (Table 1). Missing data were excluded from the analysis.

### Outcome

#### Gambling abstinence and registered date of abstinence

Users who registered as gamblers were provided with a personal counter that automatically counted the number of days since they last gambled. If they gambled, they reported it to the administrator, who resets the number of days on the counter. Those whose counters exceeded 3 years were listed on a separate website and exemplified as models that had stopped gambling. According to the list in March 2020, 161 gamblers were regarded as abstinent, without relapse for at least 3 years, and received 91,878 comments; the other 928 gamblers, who were regarded as non-abstinent, received 177,439 comments. Furthermore, these counters indicated the date when the abstinent gamblers achieved continuous 3-year abstinence. The data were also used to identify members of the self-help forum who had remained abstinent for the longest time. The counters and lists were created by the administrator without the involvement of the author of the current study.

### Independent variables

#### Social distance

All the comments involving other gamblers’ usernames were identified as comments directed at them. However, numerals, postscripts, and the word “smile” were excluded because they were frequently used in daily chats, making it difficult to extract usernames with high accuracy. If one gambler had left a comment for another gambler in a self-help forum, a direct path was drawn between them, representing a distance 1 relationship. An indirect path from one gambler to another via another gambler represented a distance 2 relationship. For example, if A comments on B and B comments on C, and A and C do not comment on each other, the relationship between A and C is regarded as distance 2 because A and C can draw a path indirectly through B. Similarly, I identified distance 3 relationships.

#### Number of comments received during the first three years

The timestamps of all comments were used as the basis for collecting comments for each gambler over 3 years. In Step 1, the date on which the gambler first commented was determined. In Step 2, the period was limited to 3 years from that date (1095 days), and all comments received by the gambler within that period were collected. In Step 3, Steps 1 and 2 are performed for all gamblers, and the total number of comments for each user is calculated. Furthermore, to show the month-to-month changes over the 3 years, these steps were repeated, limiting the period to 1 month, 2 months, and so on, up to 36 months.

#### Categories of received comments

According to the change-talk classifier from a previous study^9^, all comments were categorized into six categories: change, general, sustain, acceptive, neutral, and rejective. The change comments were positive comments about gambling abstinence (e.g., “I want to stop gambling”), whereas the sustained comments were negative comments about gambling abstinence (e.g., “Gambling makes me happy”). The general comments were neither positive nor negative, such as a narrative unrelated to current gambling (e.g., “I am busy tomorrow”). The acceptive comments were positive comments directed at others (e.g., “You are a resourceful person”). Contrarily, the rejective comments were negative (e.g., “You are a loser”). Neutral comments, like general comments, were neither positive nor negative; they served as fillers, giving information, describing the group work structure, and asking questions (e.g., “Where do you live?”). Detailed definitions of these comments have been presented elsewhere^43,62,63^. The precision and recall scores of the change-talk classifier in this study were 0.9399 and 0.9399, respectively^9^.

## Statistical analysis

### Social contagion of gambling abstinence via abstinent gamblers

Bayesian probability was used to estimate social contagion. Within the range of Social Distance 1, a sender and their recipients among abstinent gamblers were identified. If that sender had become an abstinent gambler earlier than the recipients, their relationship was considered abstinent-contagious. Conversely, if one or both of the sender and recipient duo were not abstinent, or if the sender became an abstinent gambler later than the recipients, the relationship was considered non-contagious (Supplementary Fig. 1). Similarly, contagious relationships within the range of social distances 2 and 3 were estimated.

1 $$ P\left( {B_{t1} {|}A_{t0} } \right) = \frac{{P\left( {B_{t1} ,A_{t0} } \right)}}{{P\left( {A_{t0} } \right)}} = P\left( {B_{t1} ,A*_{t0} } \right) $$

2 $$ P\left( {C_{t2} {|}B_{t1} ,A_{t0} } \right) = \frac{{P\left( {C_{t2} ,B_{t1} ,A_{t0} } \right)}}{{P\left( {B_{t1} ,A_{t0} } \right)}} = P\left( {C_{t2} ,B*_{t1} } \right) $$

3 $$ P\left( {D_{t3} {|}C_{t2} ,B_{t1} ,A_{t0} } \right) = \frac{{P\left( {D_{t3} ,C_{t2} ,B_{t1} ,A_{t0} } \right)}}{{P\left( {C_{t2} ,B_{t1} ,A_{t0} } \right)}} = P\left( {D_{t3} ,C*_{t2} } \right) $$

In the above equations, A denotes the sender’s abstinent status. B, C, and D show the abstinence status of the recipients of the sender at social distances 1, 2, and 3, respectively. t0, t1, t2, and t3 show the date when gamblers achieved 3-year continuous abstinence, and t0 < t1 < t2 < t3. Hence, all the relationships that differed in the temporal order of these data were non-contagious. Furthermore, A* indicates that the abstinent status of a sender is true. B* and C* indicate that these recipients have an abstinent-contagious relationship with A* (Supplementary Fig. 1). In other words, when a sender’s abstinence status is false, (1) is always 0. Similarly, if B does not have any contagious relationship with A*, then (2) and (3) are always 0.

### Social contagion of gambling abstinence via comments

The sender and recipients were identified within the range of social distance 1. If these recipients became abstinent gamblers and their date of becoming abstinent gamblers was later than the third year, the sender attended the forum. Furthermore, their relationship was regarded as abstinent-contagious. In contrast, if the recipient was not an abstinent gambler or if the recipient became an abstinent gambler earlier than the third year, the sender had attended the forum, the relationship was considered non-contagious (Supplementary Fig. 2). Similarly, contagious relationships within the range of social distances 2 and 3 were estimated.

4 $$ P\left( {B_{t1} {|}\overrightarrow {AB}_{t0t1} } \right) = \frac{{P\left( {B_{t1} ,\overrightarrow {AB}_{t0t1} } \right)}}{{P\left( {\overrightarrow {AB}_{t0t1} } \right)}} = P\left( {B_{t1} ,\overrightarrow {AB} *_{t0t1} } \right) $$

5 $$ P\left( {C_{t2} {|}\overrightarrow {BC}_{t1t2} ,\overrightarrow {AB}_{t0t1} } \right) = \frac{{P\left( {C_{t2} ,\overrightarrow {BC}_{t1t2} ,\overrightarrow {AB}_{t0t1} } \right)}}{{P\left( {\overrightarrow {BC}_{t1t2} ,\overrightarrow {AB}_{t0t1} } \right)}} = P\left( {C_{t2} ,\overrightarrow {BC} *_{t1t2} } \right) $$

6 $$ P\left( {D_{t3} {|}\overrightarrow {CD}_{t2t3} ,\overrightarrow {BC}_{t1t2} ,\overrightarrow {AB}_{t0t1} } \right) = \frac{{P\left( {D_{t3} ,\overrightarrow {CD}_{t2t3} ,\overrightarrow {BC}_{t1t2} ,\overrightarrow {AB}_{t0t1} } \right)}}{{P\left( {\overrightarrow {CD}_{t2t3} ,\overrightarrow {BC}_{t1t2} ,\overrightarrow {AB}_{t0t1} } \right)}} = P\left( {D_{t3} ,\overrightarrow {CD} *_{t2t3} } \right) $$

In the above equations, $\overrightarrow {AB}$, $\overrightarrow {BC}$, and $\overrightarrow {CD}$ represent comments A to B, B to C, and C to D, respectively. $\overrightarrow {AB} *$, $\overrightarrow {BC*}$, and $\overrightarrow {CD}$* indicate that these recipients have had contagious relationships with A* (Supplementary Fig. 2). In other words, if B, C, and D do not have any contagious relationship with A*, then (4), (5), and (6) are always 0.

### Partial least squares

To predict abstinent gamblers from their demographic variables, gambling problems, peers, and received comments in online self-help forums, I used the univariate partial least squares (PLS) approach because PLS is robust against correlated independent variables^64^. Furthermore, to evaluate the prediction accuracy, the area under the receiver operating characteristic curve (AUC) was used because the ratio of abstinent to non-abstinent gamblers was unbalanced^65^. Moreover, to determine the number of PLS components, we used a tenfold cross-validation approach for each PLS component from 1 to 40, as in previous studies^66^.

### Local interpretable model agnostic explanations for time series data

To determine what features predict abstinent gamblers at what time during the 36 months of forum participation, we used the Local Interpretable Model agnostic Explanations (LIME). LIME is useful for interpreting the predictive importance^67^ and can be applied to the time-series dataset^68^. For the prediction of abstinent gamblers from the four features in the forums from the first month to the 36th month (4 × 36), the K-nearest neighbor algorithm (KNN) was used because it is robust against the time-series dataset^69^. Since I used univariate LIME, four features of time-series data at 36-time points were transformed into one feature of time series data at 144-time points. Furthermore, whole features (144) were selected for LIME rather than the top 10 important features because both results were almost similar.

### Simulation network

The above two methods (PIL and LIME) assume that individuals can move freely through social networks, but in reality, they often cannot^70^. Therefore, I also examined which characteristics in a social network are useful for deterring gambling abstinence when individuals are fixed in the network through a simulation^71–74^. Simulation networks were created in which the number of comments, abstinence status, the first date of participation, and the first record pertaining to abstinence were randomly assigned, while the network structure and number of peers were fixed. One thousand simulation networks were created, and the Bayesian probabilities of being an abstinent gambler were calculated. These probabilities in the simulated networks can be regarded as mutually independent and normally distributed^71–74^. Hence, their 95% confidence intervals were estimated, and it was determined whether the observed probabilities of an abstinent gambler in actual networks were above the confidence intervals in simulated networks. The analysis was performed on Python 3, while NetworkX 2.5 was used for social network analysis.

## Ethical considerations

This study was approved by the ethics committee of the Graduate School of Technology, Industrial and Social Science, Tokushima University, Japan (reception number 222). All procedures were conducted per the guidelines for studies involving human participants, the ethical standards of the institutional research committee, and the revised 1964 Helsinki Declaration and its later amendments or comparable ethical standards. Informed consent was not obtained in our study because it is an analysis of web data and does not directly include human subjects, although it does use text written by human subjects.

## Results

### Differences in demographic variables, gambling problems, peers, and comments between abstinent and non-abstinent gamblers in the first three years of attending online self-help forums to quit gambling

The number of participants (n = 1089) exceeded the ideal sample size (n = 440). An effect size greater than 0.31 could show satisfactory power (0.9523) with alpha (0.05) in the 1089 participants. Table 1 shows the differences in demographics and gambling symptoms between the abstinent and non-abstinent gamblers. The abstinent gamblers were significantly younger than the non-abstinent gamblers, although the effect size was small. Abstinent gamblers had more gambling symptoms, especially preoccupation with gambling and lies associated with it than non-abstinent gamblers. However, they were less likely to engage in illegal activities because of gambling than non-abstinent gamblers (Table 1). These significant differences did not have sufficient power (alpha < 0.95), except for lies associated with gambling (d = 0.34 over 0.31). Additionally, it indicated that differences in demographics and gambling symptoms between abstinent and non-abstinent gamblers were limited.

Table 1 also shows the differences in comments received between abstinent and non-abstinent gamblers during the first 3 years of attending the forums. Abstinent gamblers had significantly more peers who directed comments at them in self-help forums than non-abstinent gamblers (d = 0.75). Similarly, abstinent gamblers received significantly more comments on self-help forums than non-abstinent gamblers (d = 0.58), regardless of the comment type. The effect size of these differences showed adequate power (alpha > 0.95), except for general and neutral comments. Furthermore, it indicated that abstinent gamblers had many peers from whom they received several comments in online self-help forums, as compared to non-abstinent gamblers, at a medium or higher effect size.

### Prediction of abstinent gambler from demographic variables, gambling problems, peers and received comments

To control for many positive correlations among the variables in Table 1 (Supplementary Fig. 3), PIL was used to predict abstinent gamblers because it was robust against correlated independent variables^64^. The best number of PIL components was estimated to be four through 10-cross-validation (Supplementary Fig. 4). Based on this finding, we predicted abstinent gamblers through PIL using four components. The AUC of the PIL for the entire dataset was 0.701, and the results are shown in Table 2. Table 2 shows that the number of peers in forums was a significant positive predictor of abstinent gamblers (adjusted odds ratio = 1.09), whereas the number of senior abstinent peers was not. Moreover, the number of rejective comments was a significant negative predictor of abstinent gamblers (adjusted odds ratio = 0.93), whereas the total number of comments was not. Interestingly, the disclosure of lies associated with gambling in forums was also a significant positive predictor of abstinent gamblers (adjusted odds ratio = 1.03). Although a simple comparison between abstinent and non-abstinent gamblers revealed many significant variables (Table 1), the number of valid variables was limited when the correlations among them were adjusted (Table 2). Increasing peers and decreasing rejective comments in forums could be key features of abstinent gamblers.

**Table 2:** Predictive values on abstinent gamblers through Partial Least Squares Regression.

Independent variables	*β*	*OR*	95% CI ^a^		*t*	*p*
*Demographic variable*						
Age (years)	− 0.01	0.99	0.97	1.01	− 0.94	
Male rate	0.01	1.01	0.99	1.03	1.04	
*Gambling problem*						
Total amount of debt (million yen)	0.00	1.00	0.98	1.02	0.02	
Duration of gambling (years)	0.01	1.01	0.98	1.03	0.46	
Number of symptoms(min: 1, max: 10)	− 0.02	0.98	0.93	1.03	− 0.93	
Gambling tolerance	0.01	1.01	0.98	1.04	0.44	
Unsuccessful control over gambling	− 0.01	0.99	0.97	1.02	− 0.72	
Preoccupation with gambling	0.01	1.01	0.98	1.04	0.52	
Lies associated with gambling	0.03	1.03	1.00	1.06	1.99	*
Reliance on others to provide money	0.02	1.02	0.99	1.05	1.53	
Illegal acts for gambling	− 0.02	0.98	0.96	1.01	− 1.51	
*Characteristics of peers in forums*						
Number of peers who commented	0.08	1.09	1.04	1.14	3.49	***
Number of senior abstinent peers who commented	− 0.01	0.99	0.97	1.02	− 0.60	
*Characteristics of comments received during forums*						
Number of received comments	0.02	1.02	1.00	1.03	1.76	
Number of change comments	0.01	1.01	0.96	1.06	0.32	
Number of sustain comments	− 0.04	0.96	0.93	1.01	− 1.67	
Number of general comments	0.00	1.00	0.90	1.10	− 0.08	
Number of acceptive comments	0.04	1.04	0.94	1.14	0.70	
Number of rejective comments	− 0.07	0.93	0.88	0.98	− 2.70	**
Number of neutral comments	0.04	1.04	0.92	1.18	0.61	

The number of Partial Least Squares (PLS) components was set as four based on the simulation (Supplementary Fig. 4). The dependent variable was abstinent gamblers for 3 years (yes: 1, no: 0). All independent variables were standardized. The area under the receiver operating characteristic curve was 0.701. The coefficient of determination was 0.107. The following gambling problems were not included as independent variables because consistency between the two raters and the psychologist was low: gambling withdrawal, gambling as problem avoidance, chasing one’s gambling loss, and loss of relationships and opportunities. CI: Confidence Interval, OR: adjusted odds ratio, ^a^: The 95% confidence intervals in this study are estimates because I used a pseudo-inverse matrix rather than an inverse matrix when calculating the standard errors of the regression coefficients, *p < 0.05, **p < 0.01, ***p < 0.001.

### Comparison of predictive importance for abstinent gamblers among received comments, rejective comments, senior abstinent peers, and peers for 36 months

**Figure 2:**
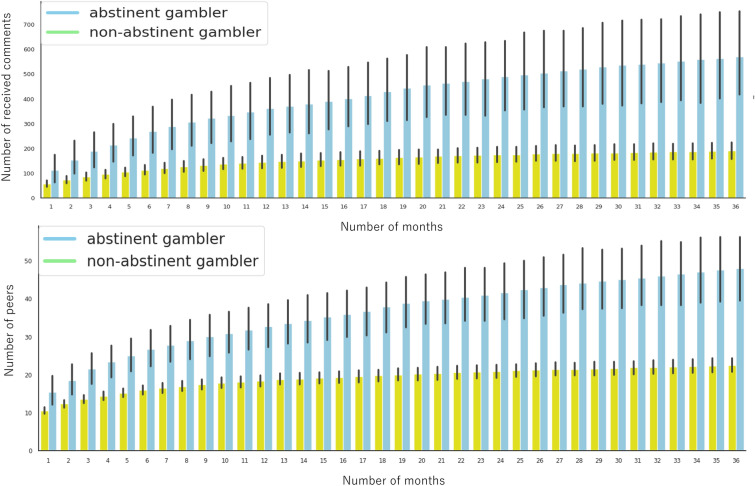
Comparison of the progress of the number of received comments and peers between abstinent and non-abstinent gamblers during the first 36 months after attending online self-help forums. The colored bars show the averages of online comments and peers between abstinent and non-abstinent gamblers. Black bars indicate the confidence intervals of these averages.

The key features of abstinent gamblers, increasing peers, and decreasing rejective comments in the forums were determined cross-sectionally in Table 2, but the optimal timing for these features to increase or decrease is still unknown. Furthermore, since the number of peers and received comments were correlated longitudinally (Fig. 2, Supplementary Table 2), the longitudinal interactive effects need to be controlled. Hence, I used the number of peers, senior abstinent peers, received comments, and rejective comments with 36-month time points as the independent variables. These four features with 36-time points were put into the KNN classifier as input data to predict abstinent gamblers. The AUC of KNN for all datasets was 0.820.

**Figure 3:**
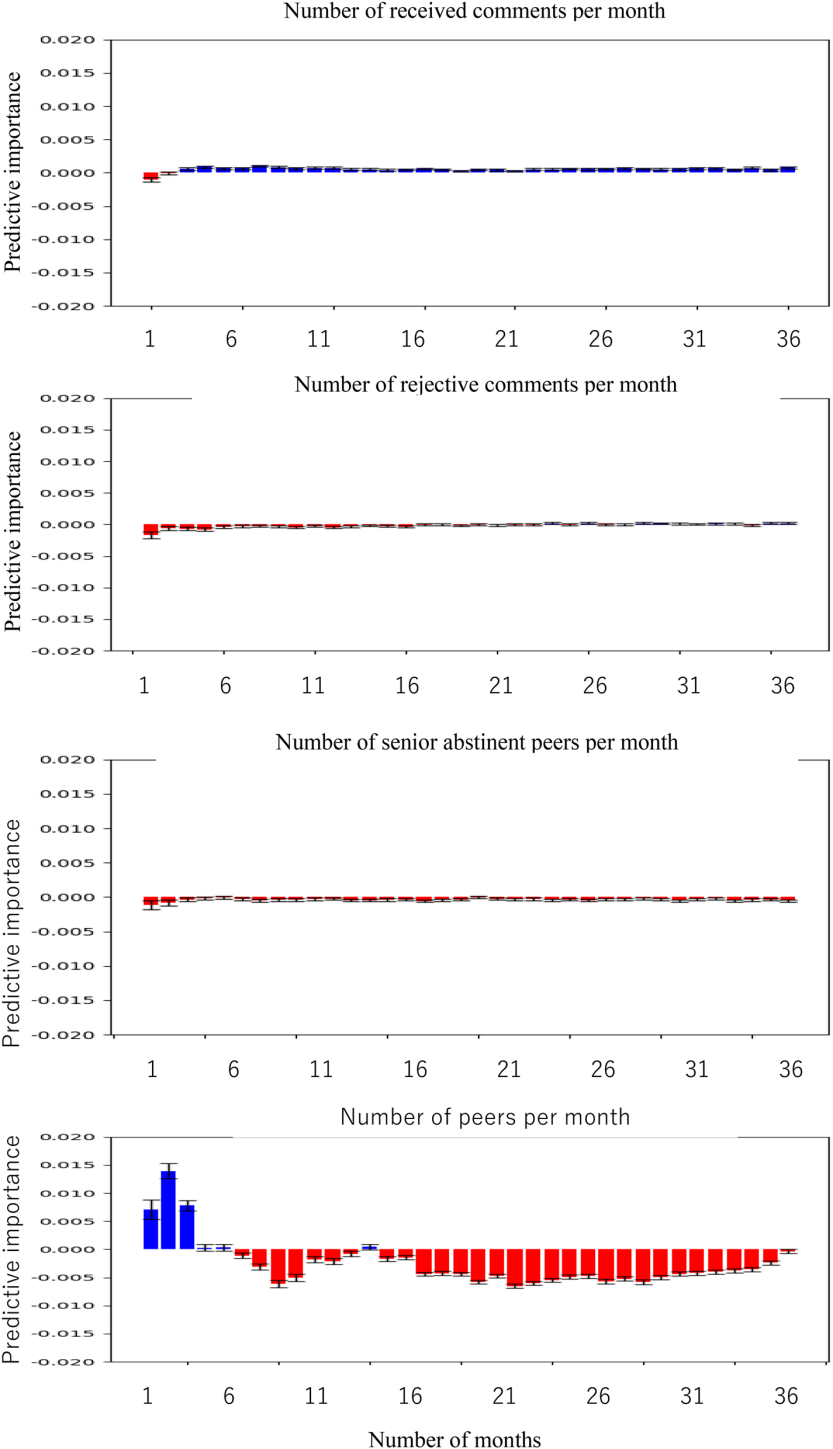
Comparison of predictive importance for abstinent gamblers among received comments, rejective comments, senior abstinent peers, and peers for 36 months. Notes: Blue and red bars indicate the positive and negative values of predictive importance in the local interpretable model-agnostic explanations (LIME). Positive values of predictive importance in a feature indicate that it positively predicts abstinent gamblers, whereas negative values in another feature indicate that it negatively predicts abstinent gamblers. The classifier used was the K-nearest neighbor algorithm (KNN), and the AUC of the KNN was 0.820.

Figure 3 shows the predictive importance of these four features and clarifies the number of peers that had the highest absolute values in these features. These findings indicated that the number of peers was the most important feature to predict abstinent gamblers. The number of peers in the first, second, and third months was a positive predictor of abstinent gamblers (Fig. 3). Contrarily, the number of peers after the 6th month was a negative predictor except for the 13th month (Supplementary Table 3). These findings indicate that a good strategy for gamblers is to make peers in the forums in the first 3 months, but after that, they do not increase the number of peers they have.

In contrast to the number of peers, the number of comments received showed a contrasting trend. The number of comments received in the first 1–2 months was a negative predictor of abstinent gamblers. However, after 3 months, they were always positive predictors of abstinent gamblers (Supplementary Table 3). Furthermore, the number of rejective comments was a negative predictor of abstinent gamblers during the first 15 months (Supplementary Table 3). Considering the results of the number of peers, receiving many comments from certain peers and avoiding peers whose comments were rejective might be a superior strategy for gamblers after 3 months.

Surprisingly, the number of senior abstinent peers was a negative predictor of abstinent gamblers (Fig. 3). These effects cannot be explained by other variables because the correlations between the number of senior abstinent peers and the other variables were less than the correlations among the other variables (Supplementary Fig. 5). These findings imply that simply increasing the number of senior abstinent peers is counterproductive to gamblers’ abstinence and that their regular engagement with their senior abstinent peers might be important to gamblers’ abstinence.

### Social contagion of gambling abstinence via abstinent gamblers and comments in online self-help forums

**Figure 4:**
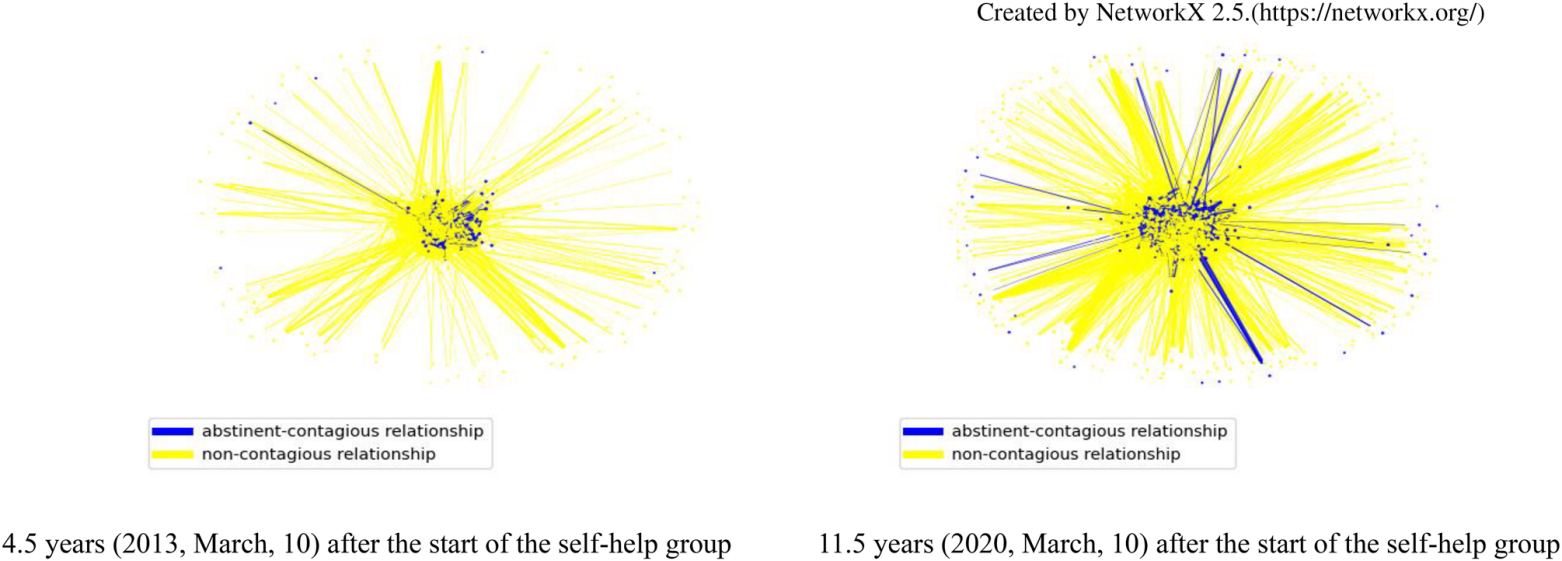
Social contagion of gambling abstinence via abstinent gamblers in the online self-help group. Blue nodes indicate abstinent gamblers who have been abstinent for at least three years, whereas yellow nodes indicate non-abstinent gamblers. The blue edges show the abstinent-contagious relationship of a gambler who became abstinent before talking to another gambler who later became abstinent. The yellow edges indicate noncontagious relationships. The number of abstinent gamblers who received comments from other gamblers 4.5 years later and 11.5 years later were 53 and 161, respectively. The number of non-abstinent gamblers who received comments from other gamblers 4.5 years later and 11.5 years later were 454 and 928, respectively. The time periods to receive comments were limited to the first three years of participation in the forums in this study.

Table 2 and Fig. 3 have confirmed that the number of peers is an important predictor of abstinent gamblers. I will now determine the features of the network that predict abstinent gamblers when the number of peers is fixed. Figure 4 shows the abstinent-contagious and non-contagious relationships in forums. Notably, the number of contagious relationships in forums increased from 2013 to 2020. This figure implies that contagious relationships have been created in forums.

**Figure 5:**
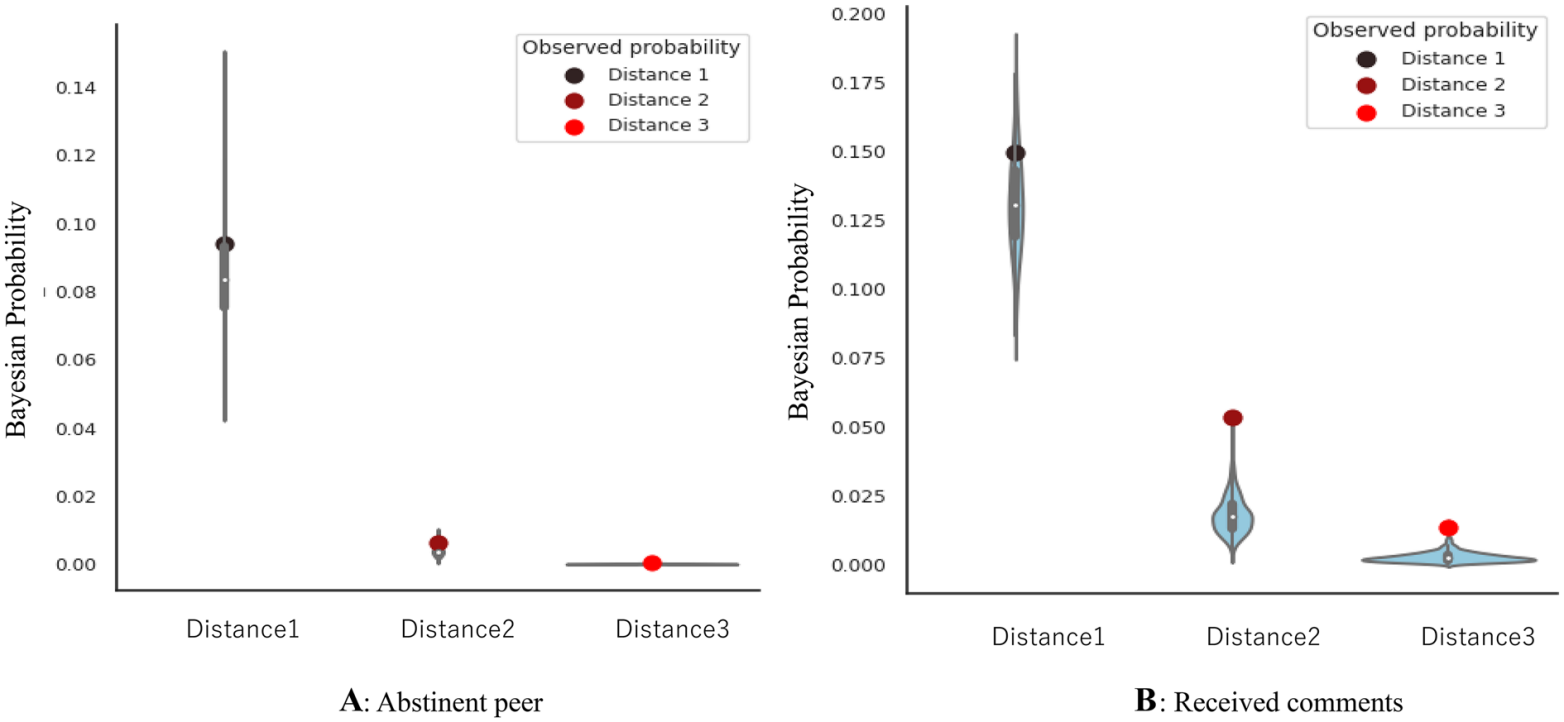
Social contagion of gambling abstinence via abstinent peers and received comments in online self-help forums. (**A**) Social contagion of gambling abstinence via abstinent peers. (**B**). Social contagion of gambling abstinence via received comments. The red dots are the observed probabilities, and the blue shapes are kernel density estimations of the underlying distribution of simulation results. The simulation results of A and B were obtained from 1000 simulations.

Figure 5A shows contagious links of senior abstinent gamblers with junior abstinent gamblers through their online peer network. It also shows that the closer the relationship with a senior abstinent gambler, which means the senior abstinent gamblers talk to the junior gambler but not others, the greater the probability that the junior gambler would also become abstinent within 3 years. The data presented in Supplementary Table 4 also support that the contagious links of senior abstinent gamblers with junior abstinent gamblers were significantly confirmed for the relationships at social distance 1 (online peer was an abstinent gambler), 2 (online peers of peers were abstinent gamblers), and 3 (online peers of peers of peers were abstinent gamblers). Corresponding with the results in Fig. 3, it indicates that it is not important for junior gamblers to have many popular abstinent peers who talk to everyone because they cannot be their close peers, but to have specific abstinent peers with a few peers because they can be their close peers (Supplementary Fig. 1). In short, the key feature of abstinent gamblers is not the number of senior abstinent peers but their closeness with senior abstinent peers.

Figure 5B also shows the contagious link of comments received through the online self-help forums with junior abstinent gamblers. Figure 5B shows that the greater the number of comments received by a gambler in the online self-help forums, the higher the probability that the gambler would become abstinent within 3 years. The data presented in Supplementary Table 4 also support that the contagious links of received comments with senior gamblers were significantly confirmed for the relationships at social distances 1, 2, and 3. Taking into account Fig. 3, the key feature of an abstinent gambler is to keep getting comments from specific peers after 3 months in the forums.

### Differences in social contagion of gambling abstinence based on comment categories

**Figure 6:**
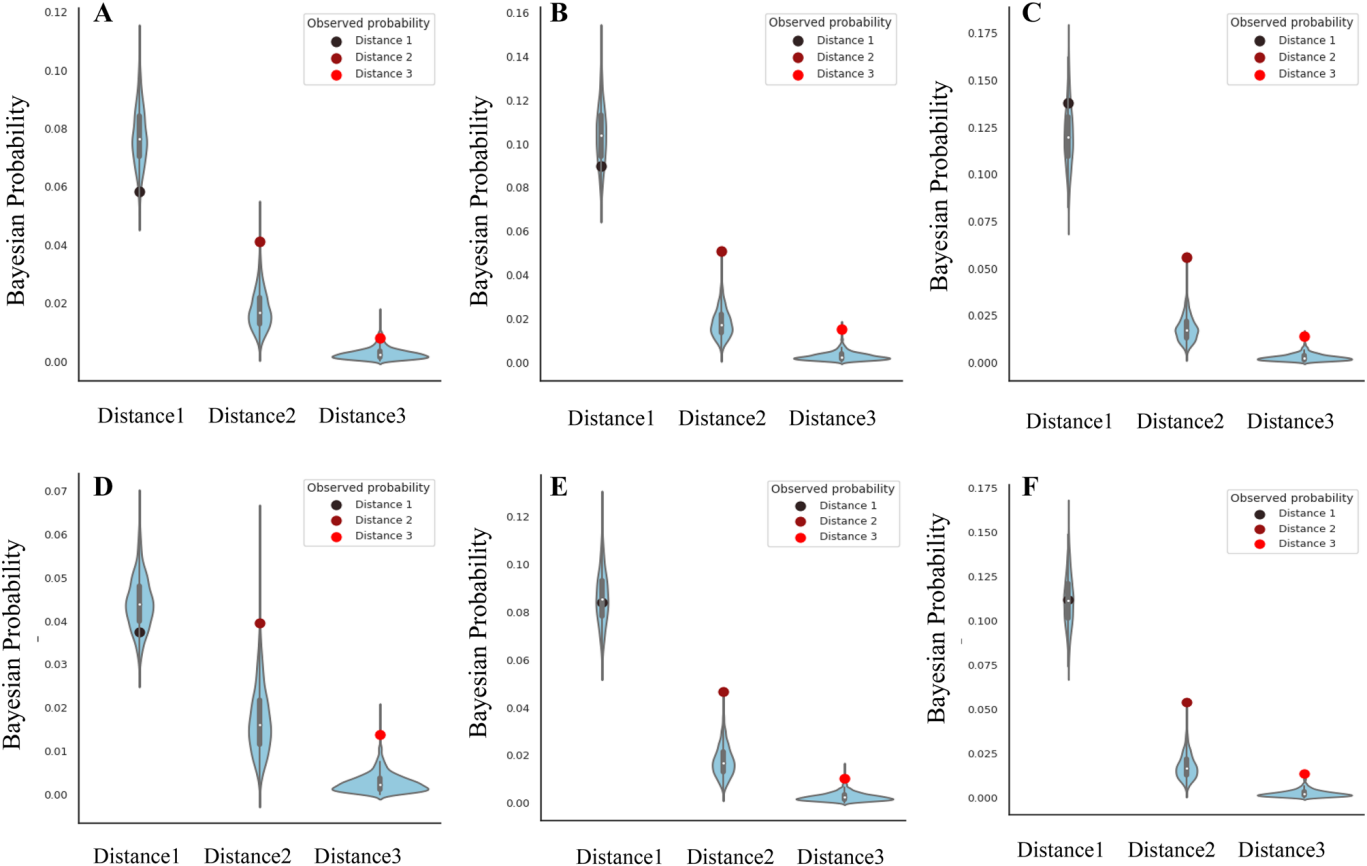
Differences in social contagion of gambling abstinence among comment categories. (**A**) Rejective comment networks, (**B**) Acceptive comment networks, (**C**). Neutral comment networks, (**D**). Sustain comment networks, (**E**). Change comment networks, (**F**). General comment networks. The red dots are the observed probabilities, and the blue shapes are kernel density estimations of the underlying distribution of simulation results. The simulation results of **A**, **B**, **C**, **D**, **E** and **F** were obtained from 1000 simulations.

I also explored differences in the social contagion of gambling abstinence based on the categories of comments in online self-help forums. Figure 6A, B, D, and E show that peers’ comments covering the rejective, acceptive, sustain, and change categories decreased the likelihood of gambling abstinence in the social distance 1 relationships but increased it in distance 2 and 3 relationships. Further, peers’ neutral comments increased the chances of gambling abstinence in social distancing 1, 2, and 3 relationships (Fig. 6C). The data presented in Supplementary Table 4 support these links in the social distance 1, 2, and 3 relationships. These results suggest that the more rejective and sustained comments they receive from close peers in the forums, the more negatively these comments affect their abstinence.

### Clinical use of social contagion of gambling abstinence via online self-help forums

Figure 2 shows the monthly progress in the number of peers between abstinent and non-abstinent gamblers during the first 36 months after attending online self-help forums. As seen in Table 2 and Fig. 3, gamblers who became abstinent after 36 months had already had more peers in the first, second, and third months than those who did not become abstinent. Supplementary Table 2 shows that abstinent gamblers received comments from an average of 15.51, 18.58, and 21.54 peers for the first, second, and third months, respectively. Conversely, non-abstinent gamblers received comments from an average of 10.57, 12.41, and 13.59 peers, respectively. Although these data had variances, it became evident that making a minimum of 15 friends in the first month and 22 friends in the third month could increase gamblers’ likelihood of quitting their habit.

Furthermore, after the fourth month, they can receive many comments from their peers (Fig. 4). Abstinent gamblers received 214.86, 242.92, and 268.91 comments on average for the fourth, fifth, and sixth months, respectively. Abstinent gamblers received approximately 30 comments per month, suggesting that they received comments from certain peers almost every day. Furthermore, frequent communication with senior abstinent peers is also effective for gambling abstinence (Fig. 5). Therefore, they must include abstinent senior peers in regular correspondence. However, peers who make rejective comments to them can have negative effects on their abstinence (Table 2, Figs. 4, 6). Thus, they need to exclude these rejective peers from regular correspondence. An objective measure shared between the clinician and gambler could help establish the latter’s monthly progress toward recovery.

## Discussion

The sample in this study comprised of gamblers who attended online self-help forums to quit their habits and clarify the links between their own abstinence and that of their peers. As expected^33–36^, having many peers for the first 3 months positively predicted gambling abstinence. When these networks were fixed, gamblers who had many comments from peers and frequent comments from senior abstinent gamblers in online self-help forums had higher chances of achieving gambling abstinence than those who did not. These findings indicate that there are two stages of successful forum participation to quit gambling: the stage of making peers and the stage of getting along with their peers. First, in the first 3 months, gamblers need to have as many peers as possible and develop a social norm of not gambling. These social norms could be effective in reducing the risks^27–31^. These findings indicate that gamblers learned abstinence from their peers in online self-help forums. Previous research found that members with many peers in a self-help forum stayed longer^39^ and that their length of stay was positively linked with their gambling abstinence^9^. Based on current and previous findings, gamblers with many peers who try to abstain from online self-help forums may stay in the forums for longer and be encouraged to be abstinent.

In the next 4 months and beyond, gamblers need to have regular contact with certain peers and sustain the habit of quitting gambling through regular contact. These regular contacts could help maintain abstinence^75–77^.To receive many comments during online forums, members must engage with one another^32^. In particular, to establish relationships in forums, individuals need to keep directing comments to others regularly^78^. The fact that the abstinent gamblers in the current study received an average of 30 comments over the course of the fourth month means that they could have been making comments every day. In other words, active participation is necessary to receive comments from peers. Furthermore, previous studies have found that many comments from peers in online self-help forums are the main factor that encourages individuals to participate actively^33–35^. Combining the current and previous findings, it can be assumed that there is a positive relationship between gamblers’ active participation and comments from their peers in online self-help forums such as online social networks^78^. This positive relationship may lead to prolonged participation in self-help forums, facilitating recovery from gambling addiction.

Consistent with the findings of a psychotherapy-based study^42^, gamblers who received comments in the sustain and rejective categories had a lower likelihood of abstinence in online self-help forums. However, inconsistent with previous findings^41^, gamblers who received change and acceptance comments also had a lower likelihood of gambling abstinence. These findings suggest that negative comments about habits were more likely to influence their habits than positive ones^79^ or that easy habits are more likely to stick than difficult ones^22^. Of course, a simple comparison cannot be made since the current findings are based on an online and group forum structure, while the previous findings are based on an offline and individual interview structure. However, the following interpretation is possible: gamblers who received change comments from their peers could have compared their progress with these peers and felt distressed^80^, potentially increasing their risk of gambling relapse^13^. Gamblers who received acceptive comments from peers were also more likely to have already experienced failure^43^, which could include gambling relapse. To validate these interpretations, future studies should examine the link between received comments and gambling abstinence in offline self-help forums, such as Gamblers Anonymous^50^.

For best practices, academic web scraping must be ethically sensitive in terms of web crawling and data organization perspectives^81^. From the perspective of web crawling, researchers should state in the methods section that the site administrator has not prohibited web scraping in terms of use^81^, that the researcher has not accessed confidential data^53^, and that the researcher has taken care not to overload the site's server^52^. Moreover, to respect the site's stakeholders, researchers should notify the website administrator or members of their use in advance, as pointed out by the current ethics review committee and one anonymous referee. From the perspective of data organization, including the disclosure of datasets^81^, researchers should not disclose data that could reveal personal information, copyright infringement, or trade secrets. From this perspective, the current study disclosed data that were not related to user ID. However, it did not disclose data related to user ID and text data since both of them might reveal personal information or infringe the copyright of participants. Furthermore, this study disclosed the 3-year success rate of gambling abstinence and the overall number of participants in the forums because the forums were organized by a citizen group^51^. This study would not disclose them if the forums were organized by a private company, because such information might fall under the category of trade secrets. Therefore, before publishing data, researchers may delete some of the data through low- or high-pass filters to prevent the disclosure of private companies’ trade secrets^72–74^. Since there is no appropriate legal system in place for web scraping, researchers are always required to give ethical considerations to individuals and organizations^81^.

This study has four limitations. First, it relied on gamblers' self-reports as the outcome variable. Some of the abstinent gamblers might have gambled but would be unable to report so in the forums due to the pressure of their surroundings, especially for popular gamblers. Therefore, the number of genuine abstinent gamblers may be lower than the number of self-reported abstinent gamblers. Second, the current study did not use a randomized controlled design. Therefore, causality between the number of peers and gambling abstinence remains unclear. For example, gamblers with few friends may have inappropriate personality traits such as psychopathy, which may have triggered their gambling^5,13^. This may be because non-abstinent gamblers reported significantly more illegal activities than abstinent gamblers in this study. It could also be that some gamblers who were highly motivated to be abstinent are commenting lots, and therefore those gamblers are receiving many comments^32^. This causality should be verified by conducting randomized controlled experiments in the future^37^. Third, the data did not include individuals who were actively involved in gambling at the time of the study because they were unwilling to register with self-help forums^36^. Hence, while this study confirmed the link between peers’ abstinence and gamblers’ abstinence, the link between peers’ gambling habits and individuals’ gambling abstinence could not be confirmed^8^. Future research needs to include multiple online groups to understand the links between peers’ abstinence and gambling and gamblers’ abstinence. Fourth, this study relied on usernames; it is possible that multiple members used the same name or that a single member used multiple pseudonyms. Hence, the identification of anonymous members is unclear.

## Conclusion

Despite these limitations, the general principle of habit formation was relevant to gambling abstinence. Although it applies mainly to the adoption of new habits, complex contagion theory could be useful for people with problematic habits to adopt alternative abstinent habits^14,16–18^. This theory assumes that people’s problematic habits can be formed based on those of their peers and their positive comments about these habits^1,2,11,20^. If so, readoption of abstinence can occur through a similar process: individuals’ abstinence can develop from their peers’ abstinence and negative comments about habits^19,21^, although negative comments about such abstinence can hinder abstinence^44,45^. The application of the complex contagion theory to problematic habits such as gambling^49^, smoking^46^, and drinking^48^ can facilitate the creation of a network-based management approach^11,12^ and reduce the suffering of people with these habits^5,13,40^.

## Supplementary Information

Supplementary Information.

## Data Availability

Datasets not associated with IDs were disclosed on the first author’s GitHub page, whereas datasets associated with IDs were not disclosed because they might reveal personal information. Further, text data were not disclosed because these data might infringe on the copyright of gamblers and/or administrators of the website.

## Code availability

The codes were disclosed on the first author’s GitHub page. However, web scraping codes were not disclosed because these codes with minor changes, such as removing time sleep function, could easily damage the server of the website.
