# Supplementary material for: Spread of gambling abstinence through peers and comments in online self-help chat forums to quit gambling
Source: Sci Rep. 2022 Mar 7;12:3675. doi: 10.1038/s41598-022-07714-2 (PMC8901770; doi:10.1038/s41598-022-07714-2)
Supplement: Supplementary file 1 — Supplementary Information. [file 41598_2022_7714_MOESM1_ESM.docx]

**Title:**

Spread of gambling abstinence through peers’ abstinence and comments in online self-help chat forums to quit gambling

**Author name and affiliation:**

Kenji Yokotani ^a*^

^a^ Graduate School of Sciences and Technology for Innovation, Tokushima University, Tokushima, Japan

**Data Availability**

Datasets not associated with IDs were disclosed on the first author’s GitHub page, whereas datasets associated with IDs were not disclosed because they might reveal personal information. Further, text data were not disclosed because these data might infringe on the copyright of gamblers and/or administrators of the website.

**Code Availability**

The codes were disclosed on the first author’s GitHub page. However, web scraping codes were not disclosed because these codes with minor changes, such as removing time sleep function, could easily damage the server of the website.

2015/5/1

2013/5/1

C1

B1

2012/5/1

C2

2011/5/1

C3

A

B2

2016/5/1

C4

B3

C5

Time t0 t1 t2

Distance 1 Distance 2

**Supplementary Figure 1. Definition of abstinent-contagious relationship via senior abstinent gamblers**

Blue faces indicate abstinent gamblers. The dates indicate when they achieved three-year continuous abstinence. White faces indicate non abstinent gamblers. The arrow indicates that the comment was made by the speaker at the start point to the listener at the endpoint. The solid blue arrow indicates an abstinent-contagious relationship, and the dashed black arrow indicates a non-contagious relationship. For a relationship to be abstinent-contagious, the speaker’s date of becoming an abstinent gambler must be earlier than the listener’s date of becoming an abstinent gambler. In the above example, the Bayesian probability that Bs will be abstinent if A is abstinent is 1/3 P(B_t1_|A_t0_). Similarly, the Bayesian probability that Cs will be abstinent if A and Bs are abstinent is 1/5: P(C_t2_|B  _t1_,A _t0_).

20

20

2015/5/1

2013/5/1

2015/5/1

C1

B1

50

2012/5/1

C2

2015/5/1

2011/5/1

20

10

C3

A

B2

20

40

2013/5/1

2016/5/1

C4

B3

20

2016/5/1

C5

Time t0 t1 t2

Distance 1 Distance 2

**Supplementary Figure 2. Definition of abstinent-contagious relationship via received comments**

Blue faces indicate abstinent gamblers. White faces indicate non abstinent gamblers. The dates indicate when they first attended the forums. The arrow indicates that the comment was made by the speaker at the start point to the listener at the end point. The numbers corresponding to each arrow indicate the number of comments from the speaker to the listener. The solid blue arrow indicates an abstinent-contagious relationship, and the dashed black arrow indicates a non-contagious relationship. For a relationship to be abstinent-contagious, the date the speaker attended their first forum needs to be earlier than that of the listener. Furthermore, the listener must be an abstinent gambler. In the above example, the Bayesian probability that Bs will be abstinent if A posts comments for Bs is 50/100: $P\left( B_{t1} | \vec{AB}_{t0t1} \right)$. Similarly, the Bayesian probability that Cs will be abstinent if A posts comments for Bs and Bs posts comments for Cs is 20/100: $P\left( C_{t2} | \vec{BC}_{t1t2},\vec{AB}_{t0t1} \right)$.


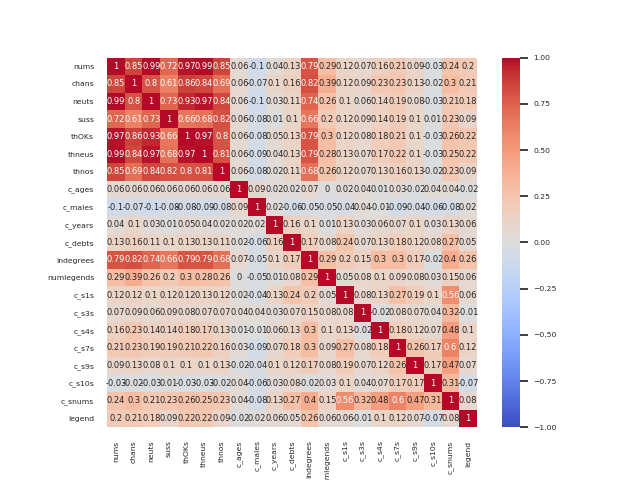


Created by seaborn 0.11.2.( https://seaborn.pydata.org/)

Received comments

Change comments

General comments

Sustain comments

Acceptive comments

Rejective comments

Neutral comments

Age

Male

Length of gambling

Debt of gambling

Peers

Abstinent peers

Gambling tolerance

Unsuccessful control

Preoccupation

Lies

Reliance

Illegal acts

Number of symptoms

Abstinence

**Supplementary Figure 3. Correlations matrix among demographic variables, gambling problems, peers and received comments**

Notes: The order of the indices in the columns is the same as the order of the indices in the rows.


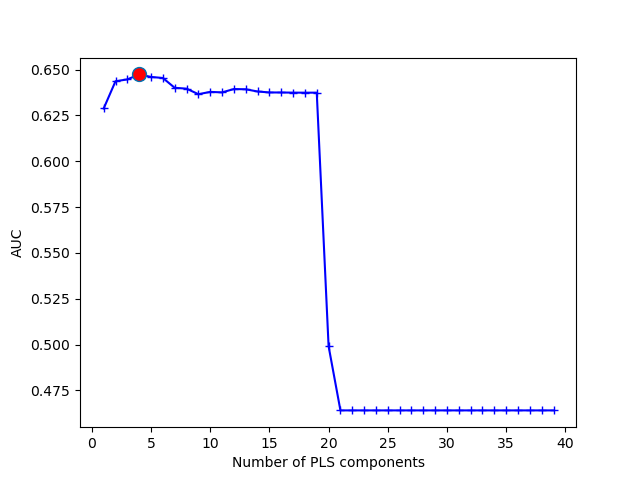


**Supplementary Figure 4. Best number of** **Partial Least Squares components through 10-cross validation**

*Notes*: AUC, area under the receiver operating characteristic curve. PLS: Partial least squares. The red circle indicates the best AUC score (0.6474) through a 10-cross validation. AUC scores of PIL from 1 to 40 components were 0.6288, 0.6437, 0.6447, 0.6474, 0.6460, 0.6454, 0.6401, 0.6396, 0.6366, 0.6379, 0.6376, 0.6394, 0.6393, 0.6381, 0.6376, 0.6375, 0.6375, 0.6374, 0.6374, 0.4991, 0.4641, 0.4641, 0.4641, 0.4641, 0.4641, 0.4641, 0.4641, 0.4641, 0.4641, 0.4641, 0.4641, 0.4641, 0.4641, 0.4641, 0.4641, 0.4641, 0.4641, 0.4641, and 0.4641, respectively.


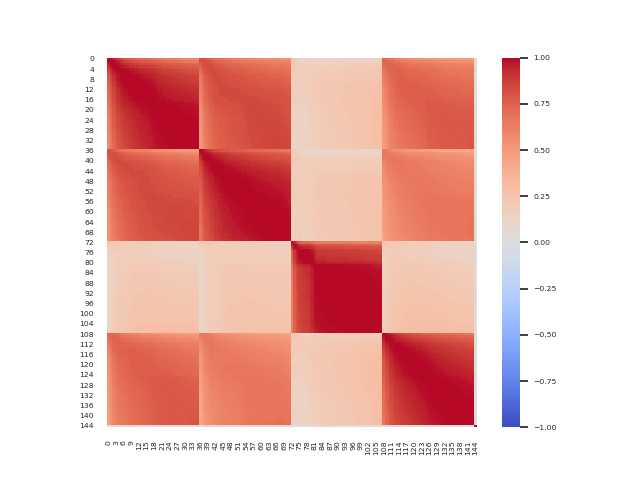


Created by seaborn 0.11.2.( https://seaborn.pydata.org/)

Abstinence (144)

Number of peers from 1^st^ month to 36^th^ month(108-143)

Number of senior abstinent peers from 1^st^ month to 36^th^ month(72-107)

Number of received comments from 1^st^ month to 36^th^ month(0-35)

Number of rejective comments from 1^st^ month to 36^th^ month(36-71)

**Supplementary Figure 5. Correlations matrix among time series variables of the number of received comments, rejective comments, senior abstinent peers, and peers for 36 months in online forums to quit gambling.**

Notes: The order of the indices in the columns is the same as the order of the indices in the rows.

**Supplementary Table 1. Concordance rates of gambling symptoms between experts and raters**

|  | Rater A | Rater B | Criterion |
| --- | --- | --- | --- |
| No gambling symptoms | 0.835 | 0.860 | ** |
| Gambling tolerance | 0.730 | 0.797 | * |
| Gambling withdrawal | 0.529 | 0.508 |  |
| Unsuccessful control over gambling | 0.799 | 0.818 | * |
| Preoccupation with gambling | 0.667 | 0.726 | * |
| Gambling as problem avoidance | 0.586 | 0.656 |  |
| Chasing one’s gambling loss | 0.735 | 0.138 |  |
| Lies associated with gambling | 0.805 | 0.777 | * |
| Loss of relationships and opportunities | 0.492 | 0.451 |  |
| Reliance on others to provide money | 0.651 | 0.884 | * |
| Illegal acts for gambling | 0.658 | 0.795 | * |

*Notes:* All scores indicate kappa coefficients. **: The agreements between the expert and two raters were over 0.80 (almost perfect agreement). *: The agreements between the expert and the two raters were over 0.60 (substantial agreement). This table is the same as that in a previous study(Yokotani, K. (2021). A change talk model for abstinence based on web-based anonymous gambler chat meeting data using an automatic change talk classifier: Development study. Journal of Medical Internet Research, 23(6), e24088.) and can be downloaded by clicking on "Supplementary Data" on the following website (<https://www.jmir.org/2021/6/e24088>).

**Supplementary Table 2. Comparison of the progress of the number of peers and received comments between abstinent and non-abstinent gamblers in the 36 months after attending online self-help forums**

| Month | Number of received comments | | | | Number of peers | | | |
| --- | --- | --- | --- | --- | --- | --- | --- | --- |
|  | Abstinent gamblers | | Non-abstinent gamblers | | Abstinent gamblers | | Non-abstinent gamblers | |
|  | *M* | *SD* | *M* | *SD* | *M* | *SD* | *M* | *SD* |
| 1 | 112.99 | 368.27 | 58.32 | 204.96 | 15.51 | 23.65 | 10.57 | 13.57 |
| 2 | 153.32 | 418.83 | 74.11 | 228.98 | 18.58 | 25.34 | 12.41 | 15.03 |
| 3 | 189.20 | 452.25 | 86.46 | 252.84 | 21.54 | 26.61 | 13.59 | 16.25 |
| 4 | 214.86 | 480.76 | 96.33 | 274.24 | 23.39 | 27.76 | 14.47 | 17.25 |
| 5 | 242.92 | 521.63 | 105.34 | 294.82 | 25.02 | 29.21 | 15.22 | 18.23 |
| 6 | 268.91 | 570.36 | 113.46 | 315.44 | 26.83 | 30.73 | 15.97 | 19.01 |
| 7 | 288.33 | 615.29 | 120.77 | 333.98 | 27.87 | 31.69 | 16.56 | 19.71 |
| 8 | 307.65 | 651.86 | 126.62 | 349.78 | 29.03 | 33.04 | 17.00 | 20.20 |
| 9 | 322.10 | 675.67 | 131.97 | 362.84 | 30.08 | 34.31 | 17.46 | 20.84 |
| 10 | 334.31 | 697.46 | 136.98 | 376.71 | 30.90 | 35.40 | 17.82 | 21.40 |
| 11 | 347.68 | 720.95 | 140.66 | 385.16 | 31.82 | 36.48 | 18.10 | 21.84 |
| 12 | 361.40 | 741.73 | 144.35 | 395.42 | 32.79 | 37.21 | 18.44 | 22.39 |
| 13 | 370.87 | 757.81 | 147.84 | 406.38 | 33.57 | 37.94 | 18.75 | 22.95 |
| 14 | 380.18 | 773.85 | 150.61 | 415.78 | 34.38 | 38.76 | 18.96 | 23.31 |
| 15 | 390.09 | 786.56 | 153.77 | 427.53 | 35.23 | 39.43 | 19.21 | 23.76 |
| 16 | 400.70 | 797.24 | 155.88 | 433.92 | 35.98 | 40.17 | 19.39 | 24.04 |
| 17 | 414.45 | 813.58 | 159.01 | 441.76 | 36.72 | 40.94 | 19.63 | 24.40 |
| 18 | 430.38 | 840.08 | 161.75 | 448.01 | 37.89 | 42.47 | 19.82 | 24.69 |
| 19 | 443.70 | 863.30 | 163.72 | 453.69 | 38.81 | 43.81 | 20.02 | 24.97 |
| 20 | 456.44 | 885.51 | 166.02 | 458.98 | 39.57 | 44.69 | 20.22 | 25.21 |
| 21 | 462.82 | 903.22 | 169.03 | 466.63 | 39.90 | 45.52 | 20.45 | 25.50 |
| 22 | 471.03 | 922.45 | 171.09 | 471.51 | 40.44 | 46.22 | 20.64 | 25.74 |
| 23 | 480.49 | 943.92 | 172.79 | 475.41 | 41.00 | 47.21 | 20.82 | 25.94 |
| 24 | 489.99 | 965.93 | 174.56 | 479.65 | 41.68 | 48.31 | 20.98 | 26.22 |
| 25 | 497.94 | 980.30 | 175.93 | 482.73 | 42.40 | 49.09 | 21.14 | 26.52 |
| 26 | 505.01 | 995.75 | 178.71 | 488.81 | 43.02 | 49.96 | 21.29 | 26.80 |
| 27 | 513.22 | 1016.89 | 179.80 | 491.48 | 43.73 | 50.75 | 21.40 | 26.97 |
| 28 | 520.64 | 1034.06 | 180.58 | 493.53 | 44.23 | 51.29 | 21.51 | 27.20 |
| 29 | 528.64 | 1054.03 | 182.09 | 501.18 | 44.73 | 51.83 | 21.64 | 27.35 |
| 30 | 535.69 | 1071.67 | 182.95 | 503.18 | 45.07 | 52.28 | 21.75 | 27.49 |
| 31 | 540.44 | 1079.56 | 184.37 | 506.13 | 45.47 | 52.65 | 21.92 | 27.74 |
| 32 | 545.37 | 1085.69 | 185.59 | 508.77 | 45.98 | 53.27 | 22.02 | 27.87 |
| 33 | 552.03 | 1097.57 | 187.11 | 512.26 | 46.61 | 54.06 | 22.15 | 28.05 |
| 34 | 558.73 | 1117.19 | 188.45 | 515.96 | 47.11 | 54.84 | 22.25 | 28.23 |
| 35 | 563.96 | 1130.39 | 189.68 | 518.49 | 47.62 | 55.49 | 22.38 | 28.41 |
| 36 | 570.67 | 1142.71 | 191.21 | 521.42 | 47.98 | 56.02 | 22.54 | 28.65 |

*Notes.* M: mean, SD: standard deviation.

**Supplementary Table 3. Comparison of the predictive importance of abstinent gamblers among increases in received comments, rejective comments, senior abstinent peers, and peers in online forums to quit gambling.**

| Month | Received comments | | Rejective comments | | Senior abstinent peers | | Peers | |
| --- | --- | --- | --- | --- | --- | --- | --- | --- |
|  | *M* | *SE* | *M* | *SE* | *M* | *SE* | *M* | *SE* |
| 1 | -0.0010 | 0.0003 | -0.0017 | 0.0005 | -0.0012 | 0.0006 | 0.0071 | 0.0017 |
| 2 | -0.0001 | 0.0002 | -0.0006 | 0.0003 | -0.0008 | 0.0004 | 0.0139 | 0.0014 |
| 3 | 0.0006 | 0.0002 | -0.0007 | 0.0003 | -0.0004 | 0.0003 | 0.0078 | 0.0009 |
| 4 | 0.0009 | 0.0002 | -0.0008 | 0.0002 | -0.0002 | 0.0002 | 0.0003 | 0.0006 |
| 5 | 0.0006 | 0.0002 | -0.0004 | 0.0002 | -0.0001 | 0.0002 | 0.0003 | 0.0006 |
| 6 | 0.0007 | 0.0002 | -0.0003 | 0.0002 | -0.0003 | 0.0002 | -0.0011 | 0.0005 |
| 7 | 0.0010 | 0.0002 | -0.0002 | 0.0002 | -0.0005 | 0.0002 | -0.0031 | 0.0005 |
| 8 | 0.0008 | 0.0002 | -0.0003 | 0.0002 | -0.0004 | 0.0002 | -0.0061 | 0.0007 |
| 9 | 0.0007 | 0.0001 | -0.0005 | 0.0002 | -0.0004 | 0.0002 | -0.0050 | 0.0007 |
| 10 | 0.0007 | 0.0001 | -0.0002 | 0.0002 | -0.0003 | 0.0002 | -0.0018 | 0.0006 |
| 11 | 0.0007 | 0.0002 | -0.0005 | 0.0002 | -0.0002 | 0.0002 | -0.0022 | 0.0005 |
| 12 | 0.0005 | 0.0001 | -0.0003 | 0.0002 | -0.0004 | 0.0002 | -0.0008 | 0.0005 |
| 13 | 0.0005 | 0.0001 | -0.0002 | 0.0002 | -0.0005 | 0.0002 | 0.0004 | 0.0005 |
| 14 | 0.0004 | 0.0001 | -0.0002 | 0.0001 | -0.0004 | 0.0002 | -0.0017 | 0.0004 |
| 15 | 0.0005 | 0.0001 | -0.0003 | 0.0002 | -0.0004 | 0.0002 | -0.0014 | 0.0004 |
| 16 | 0.0006 | 0.0001 | 0.0000 | 0.0002 | -0.0006 | 0.0002 | -0.0044 | 0.0003 |
| 17 | 0.0005 | 0.0001 | 0.0000 | 0.0001 | -0.0004 | 0.0002 | -0.0042 | 0.0003 |
| 18 | 0.0002 | 0.0001 | -0.0002 | 0.0001 | -0.0003 | 0.0002 | -0.0044 | 0.0003 |
| 19 | 0.0005 | 0.0001 | 0.0000 | 0.0002 | 0.0000 | 0.0002 | -0.0058 | 0.0004 |
| 20 | 0.0004 | 0.0001 | -0.0001 | 0.0002 | -0.0003 | 0.0002 | -0.0047 | 0.0003 |
| 21 | 0.0003 | 0.0001 | -0.0001 | 0.0002 | -0.0003 | 0.0002 | -0.0065 | 0.0004 |
| 22 | 0.0005 | 0.0001 | 0.0000 | 0.0002 | -0.0003 | 0.0002 | -0.0060 | 0.0004 |
| 23 | 0.0005 | 0.0001 | 0.0002 | 0.0001 | -0.0004 | 0.0002 | -0.0054 | 0.0003 |
| 24 | 0.0006 | 0.0001 | 0.0000 | 0.0001 | -0.0004 | 0.0002 | -0.0049 | 0.0003 |
| 25 | 0.0006 | 0.0001 | 0.0002 | 0.0002 | -0.0005 | 0.0002 | -0.0048 | 0.0003 |
| 26 | 0.0006 | 0.0001 | 0.0000 | 0.0002 | -0.0003 | 0.0002 | -0.0057 | 0.0004 |
| 27 | 0.0007 | 0.0001 | 0.0000 | 0.0002 | -0.0003 | 0.0002 | -0.0052 | 0.0004 |
| 28 | 0.0006 | 0.0001 | 0.0002 | 0.0001 | -0.0002 | 0.0002 | -0.0058 | 0.0004 |
| 29 | 0.0005 | 0.0001 | 0.0001 | 0.0001 | -0.0003 | 0.0002 | -0.0050 | 0.0004 |
| 30 | 0.0006 | 0.0001 | 0.0001 | 0.0001 | -0.0005 | 0.0002 | -0.0043 | 0.0004 |
| 31 | 0.0007 | 0.0001 | 0.0000 | 0.0002 | -0.0003 | 0.0002 | -0.0042 | 0.0004 |
| 32 | 0.0007 | 0.0001 | 0.0001 | 0.0002 | -0.0002 | 0.0002 | -0.0040 | 0.0004 |
| 33 | 0.0005 | 0.0001 | 0.0001 | 0.0002 | -0.0005 | 0.0002 | -0.0038 | 0.0004 |
| 34 | 0.0007 | 0.0001 | -0.0002 | 0.0002 | -0.0004 | 0.0002 | -0.0035 | 0.0004 |
| 35 | 0.0004 | 0.0001 | 0.0002 | 0.0002 | -0.0004 | 0.0002 | -0.0024 | 0.0004 |
| 36 | 0.0007 | 0.0002 | 0.0002 | 0.0002 | -0.0005 | 0.0002 | -0.0004 | 0.0004 |

*Notes:* M, mean; SE, standard error. Predictive importance was calculated by LIME (local interpretable model-agnostic explanations). Positive values of predictive importance in a feature indicate that it positively predicts abstinent gamblers, whereas negative values in another feature indicate that it negatively predicts abstinent gamblers.

**Supplementary Table 4. Effects of peers’ abstinence and received comments on gambling abstinence in online self-help networks**

| Simulation data | |  |  |  | Observed data | |
| --- | --- | --- | --- | --- | --- | --- |
| Relationship | Network category | Average of 95% CI | Lower limit of 95% CI | Upper limit of 95% CI | Bayesian probability | p |
| Distance 1 | Abstinent gambler | 0.0840 | 0.0832 | 0.0849 | 0.0939 | * |
| Distance 2 | Abstinent gambler | 0.0040 | 0.0039 | 0.0041 | 0.0064 | * |
| Distance 3 | Abstinent gambler | 0.0002 | 0.0002 | 0.0002 | 0.0006 | * |
| Distance 1 | Number of comments | 0.1311 | 0.1300 | 0.1322 | 0.1496 | * |
| Distance 2 | Number of comments | 0.0181 | 0.0177 | 0.0185 | 0.0533 | * |
| Distance 3 | Number of comments | 0.0028 | 0.0027 | 0.0029 | 0.0137 | * |
| Distance 1 | Change comments | 0.0858 | 0.0852 | 0.0865 | 0.0841 | * |
| Distance 2 | Change comments | 0.0178 | 0.0174 | 0.0182 | 0.0468 | * |
| Distance 3 | Change comments | 0.0028 | 0.0026 | 0.0029 | 0.0104 | * |
| Distance 1 | Sustain comments | 0.0441 | 0.0437 | 0.0445 | 0.0375 | * |
| Distance 2 | Sustain comments | 0.0171 | 0.0167 | 0.0176 | 0.0396 | * |
| Distance 3 | Sustain comments | 0.0028 | 0.0026 | 0.0029 | 0.0137 | * |
| Distance 1 | General comments | 0.1116 | 0.1107 | 0.1125 | 0.1118 |  |
| Distance 2 | General comments | 0.0180 | 0.0176 | 0.0185 | 0.0543 | * |
| Distance 3 | General comments | 0.0028 | 0.0027 | 0.0029 | 0.0139 | * |
| Distance 1 | Acceptive comments | 0.1042 | 0.1034 | 0.1050 | 0.0896 | * |
| Distance 2 | Acceptive comments | 0.0181 | 0.0177 | 0.0185 | 0.0508 | * |
| Distance 3 | Acceptive comments | 0.0028 | 0.0027 | 0.0029 | 0.0151 | * |
| Distance 1 | Rejective comments | 0.0772 | 0.0766 | 0.0779 | 0.0585 | * |
| Distance 2 | Rejective comments | 0.0179 | 0.0175 | 0.0183 | 0.0413 | * |
| Distance 3 | Rejective comments | 0.0027 | 0.0026 | 0.0028 | 0.0081 | * |
| Distance 1 | Neutral comments | 0.1203 | 0.1194 | 0.1213 | 0.1379 | * |
| Distance 2 | Neutral comments | 0.0181 | 0.0177 | 0.0185 | 0.0558 | * |
| Distance 3 | Neutral comments | 0.0028 | 0.0027 | 0.0029 | 0.0140 | * |

*Notes*: CI: confidence interval. The CI was calculated based on 1,000 simulations. ∗: p < .05.
